# Friend or foe: the gut microbiota as a modulator of disease trajectory in trauma, surgery, and critical illness

**DOI:** 10.1080/19490976.2025.2552346

**Published:** 2025-08-29

**Authors:** Jessica Cao, Rebecca Meltzer, Ellen Cohn, Andrew Benjamin, Robert Keskey, John Alverdy

**Affiliations:** Department of Surgery, University of Chicago, Chicago, IL, USA

**Keywords:** Gut microbiota, microbiota metabolites, surgery, trauma, critical illness

## Abstract

The connections between host physiology and the gut microbiome continue to grow as we learn how both the composition and function of the microbiota can impact organ systems beyond the gastrointestinal tract (e.g. the liver, lungs, kidneys, brain). An individual’s unique life history plays an important role in shaping the gut microbiota, whose composition and functional output may influence the clinical course and outcomes following a major physiologic perturbation such as surgery or trauma in a manner that extends beyond host genetics. Alterations in the gut microbiota that occur during the course of an illness may explain some of the heterogeneity seen in both recovery and the development of complications. Here, we challenge traditional perspectives on infectious complications, in which pathogenic strains of bacteria are considered to be the sole perpetrators. In this review, we instead examine the crosstalk between the pathogen, the host, and its colonizing microbiome – termed the “interactome”–and explore its role in driving disease progression or recovery following major physiologic perturbations including surgery, trauma, and critical illness.

## Introduction

Patients undergoing surgery, experiencing traumatic injury, or facing critical illness often follow vastly different clinical trajectories despite similar initial presentations or interventions. Outcomes are highly variable, as some recover uneventfully while others experience devastating infectious complications, organ failure, or prolonged ICU stays. Traditionally, this heterogeneity has been attributed to differences in host factors (e.g. comorbidities, age) or the severity of the inciting insult. However, there is growing evidence to suggest that this explanation is incomplete. Recently, the gut microbiota – and its interactions with both the host and invading pathogens – has emerged as a key player in shaping the course of disease and recovery following major physiologic stress such as trauma, surgery, and critical illness.

This shift in perspective is grounded in the concept that virulence (i.e. harmfulness, clinical infection) is best viewed not as a static property of the microbe or host alone, but rather as a property of their interaction with one another and with the host’s colonizing microbiome^1^. For instance, prior work from our group has shown that when the microbiota’s composition and function are perturbed by a combination of factors such as antibiotics, diet, and surgical stress, mice are more susceptible to developing severe multi-drug-resistant infections post-operatively despite a sterile procedure^2^. These findings suggest that dysbiosis – a disruption in the native gut microbiota – can alter the behavior of resident microbes and promote the emergence of a “pathobiome” capable of driving complications. Thus, a deeper understanding of the “interactome”–the dynamic and reciprocal dialogue between host, microbiota, and pathogens – is imperative to uncovering what drives health, disease progression, and recovery following physiologic perturbations.

Currently, the gut microbiota remains underappreciated in clinical frameworks for infection-related complications in trauma, surgical, and critical care patients. Conventional infection-related research relies on correlating host immune responses with survival following a standardized infectious insult (e.g. cecal ligation and puncture, endotoxin administration, systemic bacterial injection). However, this host-centric approach overlooks the microbiota’s role in shaping systemic immunity, facilitating organ crosstalk through downstream metabolites, and modulating host susceptibility to infectious complications. In this review, we aim to appreciate how gut microbes may act in a manner beyond lending a source of pathogenic strains. We will first briefly describe how the microbiota modulates host physiology, and then explore how trauma, surgical interventions, and critical care practices disrupt this balance, contributing to complications or hindering recovery. Finally, we consider how the concept of the host-microbiota-pathogen interactome can inform new microbiota-directed approaches to guide treatment and improve outcomes.

## The gut microbiota and host physiology

### Healthy microbiota composition and function

The healthy intestinal microbiota is composed of diverse communities of bacteria that colonize distinct areas of the gastrointestinal tract, though they primarily reside in the large intestine^3^. Dominant phyla include health-promoting gram positive *Firmicutes* and gram negative *Bacteroidetes*, with often disease-associated *Proteobacteria* present in lower concentrations^4^. While species-level composition of the gut microbiota fluctuates among individuals^5,6^–dependent on factors such as age, genetics, diet, and environment (e.g., access to healthcare, industrialization, antibiotics, C-section, physical activity), the microbiota functions collectively to support host physiology. These complex microbial communities have co-evolved with their hosts, shaped by the host’s life history^7^, and depend on one another as well as the host for basic metabolic function and survival^8,9^. These biochemical processes not only help maintain homeostasis amongst the bacterial communities, but they also have a significant impact on biologic processes within the host.

Functionally, the gut microbiota plays a vital role in host immunity and systemic health through both direct interactions and the production of microbial metabolites^10^. It fortifies mucosal integrity by promoting mucus production, stimulating antimicrobial peptide production by Paneth cells, and enhancing IgA secretion^11^. Specific bacterial components such as polysaccharide A (PSA) drive the development and balance of regulatory T cells and Th17 cells, while also contributing to the development of unconventional cell populations like iNKT and MAIT cells^12^. Additionally, microbial communities within the gut generate a wide array of bioactive metabolites, many of which modulate physiologic functions essential for host health. Anaerobic bacteria within the gut produce short chain fatty acids (SCFA) including acetate, propionate and butyrate via the fermentation of dietary carbohydrates (i.e., microbiota-accessible carbohydrates).Butyrate, in particular, acts as an HDAC inhibitor or a G-protein coupled receptor ligand and can alter macrophage response to improve bacterial clearance while reducing associated immunopathology^13,14^. Tryptophan metabolism by gut microbes yield indole metabolites that activate the ubiquitously present aryl hydrocarbon receptor (AhR), playing a role in modulating innate lymphoid cell signaling, responding to quorum sensing molecules secreted by pathogens like *Pseudomonas*, and bolstering immune responses to lethal bacterial infection^15–19^. Similarly, gut bacteria play an important role in bile acid conjugation and the production of secondary bile acids that regulate Treg cell populations, help maintain barrier integrity, and alter dendritic cell responsiveness to pathogens^20^. Whether through direct stimulation of the host immune response with gut bacterial antigens or via the production of metabolites, gut. bacteria have the ability to alter the host’s immune system both locally at the intestinal surface, but also systemically.

### The microbiota and organ crosstalk

The gut microbiota engages in continuous bidirectional communication with distant organs through immune signaling and microbial metabolites. These “gut-organ axes” are critical for maintaining homeostasis and their disruption during states of physiologic stress can help explain varying degrees of organ dysfunction seen in trauma, surgical, and critical care patients. In the gut-lung axis, microbial metabolites such as SCFAs and indoles modulate alveolar macrophage responses and cytokine responses, and their depletion during critical illness may heighten susceptibility to pulmonary infections as well as pathologies like acute respiratory distress syndrome (ARDS)^21–23^. Loss of anaerobic gut bacteria due to exposure to anti-anaerobic antibiotics, as well as exposure to hyperoxia during critical illness has been shown to precede and exacerbate pulmonary complications^24,25^. The gut-brain axis involves both neural and humoral pathways^26^, and its disruption has been implicated in a number of pathologies including ICU delirium, stress-related neuroinflammation, sequelae of traumatic brain injury, and even long-term cognitive decline following critical illness^27–29^. Additionally, dissemination of gut-origin microbes to the brain has been observed in both animal models and patients dying of sepsis and is associated with the neuroinflammation that underlies septic encephalopathy^30^. With regard to the gut-kidney axis, uremic toxins (i.e. p-cresol and indoxyl sulfates) produced by microbial fermentation accumulate in the setting of renal dysfunction and contribute to renal oxidative stress and inflammation as well as further intestinal dysbiosis as the colon becomes a compensatory site for uric acid excretion. Animal studies have demonstrated that microbiota-directed therapies such as SCFA or probiotic administration can prevent acute renal dysfunction after ischemia-reperfusion injury^31,32^. Finally, the gut-liver axis is shaped by microbial-derived bile acids and endotoxins that can enter the portal circulation that regulate immune tolerance, lipid metabolism, and hepatic detoxification^33–35^. In disease, gut dysbiosis – often triggered by factors like high-fat diets and alcohol use – impairs intestinal barrier function, allowing secreted microbial products (e.g. LPS) to enter the portal circulation and trigger hepatic inflammation^36,37^. Together, these gut-organ axes highlight the gut microbiota’s role as a systemic regulator and how dysbiosis can contribute to multi-system organ dysfunction and variable recovery trajectories following major physiologic insults.

## Impact of critical illness on the gut microbiota

As evidenced by the above discussion, it is clear the gut microbiota is crucial for maintaining host physiology, as it contributes to immune development and modulates function across various distant organ systems. This may be especially important as patients attempt to recover from a major physiologic perturbation such as traumatic injury or surgical stress. To fully appreciate how gut microbes contribute to either recovery from or progression of illness when a physiologic insult occurs, it is important to consider how the gut ecosystem shifts in composition and function in response to various host factors during the insult itself as well as during treatment. Exploring the reciprocal nature of host-pathogen interactions affords us a more holistic view of how infection-related complications arise following a catabolic stress – not simply as a result of pathogen overgrowth and dissemination, but rather via mechanisms that disrupt host-microbe homeostasis.

### Trauma and the gut microbiota

Trauma is often considered a systemic disease that can both affect multiple organ systems and trigger a widespread inflammatory response within minutes to hours of injury. Tissue damage – which includes large scale damage to external barriers (e.g. skin) and organ systems, as well as molecular and cellular grade damage – can rapidly elicit the innate immune system to address damaged tissues. The goal of this rapid and robust response is to balance pro-inflammatory and anti-inflammatory pathways, ultimately to clear tissue and molecular damage and to initiate healing mechanisms. However, as one can imagine, in cases of severe injury, extensive surgical intervention, or hemorrhagic shock, this response can often become imbalanced.

Severe injury and tissue trauma has been shown to lead to cellular disruption with subsequent release of cellular debris and molecular “danger signals,” including damage-associated molecular patterns (DAMPs; e.g. ATP, mitochondrial DNA) which are recognized by the immune system as “self,” as well as pathogen-associated molecular patterns (PAMPs) which are secreted by gut microbes and recognized as “non-self.” DAMPs and PAMPs can penetrate damaged tissues to induce additional local endothelial damage and activate the immune response systemically. Organ damage and infection can lead to further release of DAMPs and PAMPs, leading to a vicious cycle of inflammation and immune dysregulation^38,39^. This frequently affects the intestinal mucosa, and in combination with the gut dysmotility and hypoperfusion that are common in trauma patients, they can lead to increased mucosal inflammation and a shift in commensal health-promoting anaerobes to more virulent strains (e.g. *Pseudomonas*) as well as an overgrowth of *Enterococcus* species^40–44^.

Trauma can negatively impact the gut in multiple ways including by global hypoperfusion from hemorrhagic shock, interrupting the gut-brain axis in cases of traumatic brain injury, or direct injury to the gut itself^43^. In preclinical models of severe trauma, it has been demonstrated that hemorrhagic shock alone can lead to increases of opportunistic pathogens^45^. Additionally, in a rat model of hemorrhagic shock and laparotomy, there was increased intestinal permeability and a breakdown of the mucosal intestinal barrier with increased enterocyte apoptosis^46^. In murine models across multiple injury complexes including traumatic brain injury, spinal cord injury, burn injury, and polytrauma, there has been an observed decrease in gut microbial diversity as soon as 6 hours after injury, which persisted for as long as 30 days post injury. Additional studies have demonstrated a decreased abundance of butyrate-producing bacterial families^47^. Though clinical studies are more limited, serial stool monitoring has demonstrated that severely injured patients have a depletion of *Bacteriodales* species at 72 hours post injury, and an abundance of often pathogenic *Enterococcus and Clostridiales* species compared to healthy controls. Furthermore, higher microbial diversity was associated with improved survival in some studies^43,43^. This rapid and persistent dysbiosis in combination with the overwhelming inflammatory state after traumatic injury and increased intestinal permeability have been proposed to be linked to the high rates of secondary infections in trauma patients (e.g. lung, wound, intra-abdominal) with the incidence of sepsis after severe trauma reported to be as high as 50%^40^.

### Surgical stress and the gut microbiota

Major surgery is another source of physiologic stress that can result in a profound shift in the gut microbiota. In combination with other insults such as trauma or burn injury as described above, surgery can result in a major shift in the intestinal microbiota, with studies of burn injury and associated surgery demonstrating an up to 90% reduction in health promoting *Bacteriodetes* and *Firmicutes* species and dramatic increases in pathogenic *Proteobacteria* ^48^. Furthermore, surgeries involving the gastrointestinal tract result in major shifts in intestinal microbiota composition. In a rat surgical model, colon resection was shown to lead to dramatic increases in *Escherichia and Enteroccocus* species, with an associated predominance of bacterial virulence-associated pathways within the anastomotic tissues^49^. In mouse models of small bowel resection, there were significant changes in the microbial composition in the ileum, and these changes persisted for up to 90 days after surgery^50^. In addition, much of the preparation for intestinal surgery requires a fasting state, administration of prophylactic antibiotics, and even mechanical and chemical bowel preparation, all of which can shift the gut microbiota toward pathogenic species and increase susceptibility to infection^51^. Furthermore, intestinal surgery such as bowel resection or the creation of an ostomy, requires a breach of the intestinal epithelial barrier, which exposes the intestines, a normally anaerobic environment, to the outside world. This exposure, such as in patients with an ileostomy, has been demonstrated to lead to the depletion of obligate anaerobes including *Bacteroides* and the increase of facultative anaerobes such as *Enterobacteriacae* ^52^. It is also important to note that the stress of surgery itself – independent of the resection and manipulation of bowel – also affects the gut microbiota, as evidenced by studies involving non-intestinal surgeries. Depletion of SCFA-producing bacteria and increased abundance of gram negative bacteria have been reported following orthopedic surgery.^53^ After cardiac surgery, there is a decline in facultative anaerobes as well as SCFA levels with a rise in the relative abundance of *Enterobacteriaceae* and *Staphylococcus*. ^54^ Finally, our group has found that Pseudomonas harvested from the intestine of mice subjected to surgical injury in the form of a 30% hepatectomy express enhanced virulence, as reflected by their ability to cause lethal peritonitis compared to strains harvested from control mice^55^. This demonstrates that the act of surgery itself can change virulence expression, driving a shift from commensal microbiota to pathobiota.

The intestinal microbiota is an important defense mechanism against infectious complications, but in the case of surgical stress in combination with the preparation required prior to surgery, pathogenic bacteria can prevail and lead to major post-surgical complications. These complications include anastomotic leak, infection with *C. difficile*, or disseminated infection^56^. In fact, the most common cause of readmission following surgery is post-operative infection, which can be as high as 25–30% in patients undergoing a colon resection^57^, despite the many methods intended to reduce the presence of pathogens, such as prophylactic antibiotics and ensuring sterility during procedures. While previously, post-operative infectious complications were thought to be secondary to a breach in infection control techniques, they may be better understood as a consequence of disrupting the interactome or, in other words, the delicate homeostasis between the host immune system, pathogens, and the gut microbiota.

### Wound healing and the gut microbiota

Thus far, we have explored the dynamic interplay between the host, gut mirobiota, and pathogens in the context of trauma and surgical stress. These physiologic insults initiate a cascade of changes – not only within the immune system and organ systems, but also within the gut microbiota itself, driving pathoadaptive shifts that transform it from a health-promoting ecosystem into one that may harbor virulent opportunistic pathogens. One particularly tangible and clinically relevant manifestation of how the altered host-microbiota interaction in trauma and surgical patients can tip the balance between recovery and complication is wound healing.

Similar to other organs, the skin and associated integument is also influenced by the presence of its own microbiota as well as those present in the gut. Despite all attempts to make the wound environment sterile, all wounds become contaminated with microorganisms from both exogenous (e.g., from the environment) and endogenous (e.g., skin, gut flora) sources. The progression from contamination to infection is dependent upon several factors including local environment, host comorbidities, microbial virulence factors, and complex signaling within the host-microbe “interactome”^58^. Therefore, to effectively understand and address wound-healing and infection-related complications, it is essential to consider the contributions of the “wound microbiota.” Several studies have shown that within surgical incisions, there is a depletion of skin commensals and subsequent expansion of potentially pathogenic environmental microorganisms, such as *Enterococcus faecalis* and *Enterobacteriaceae* at colorectal incisions and *S. aureus* at Mohs surgical sites^59,60^. This shift in the wound microbiota may in part be secondary to surgical preparatory procedures that, while intended to prevent surgical site infection, may instead inadvertently disrupt native microbial communities and heighten susceptibility to infection^61–63^.

Further evidence for a role of the gut microbiota in wound healing can be understood by its contributions to the healing of intestinal anastomoses – surgical connections to maintain intestinal continuity following segmental resection. Our group previously demonstrated that the gut commensal, *Enterococcus faecalis*, has the capacity to degrade collagen and activate tissue matrix metalloproteinase 9 (MMP9), ultimately contributing to poor anastomotic healing and the development of anastomotic leak in rats. We demonstrated that leaking anastomotic tissues were colonized by strains of *E. faecalis* that had increased collagenase activity and ability to activate host MMP9. Conversely, elimination of *E. faecalis* at the anastomosis and administration of an MMP9 inhibitor I reduced leak rates^64^. Furthermore, we have shown in a mouse model of anastomotic leak that tranexamic acid enemas can decrease leak rates by inhibiting plasminogen activation by gut-derived bacteria at the site of colorectal anastomoses^65^.

Beyond local effects on anastomotic healing, the gut microbiota has also been shown to affect remote tissues after surgical stress and increase the risk of surgical site infections (SSI) at sites distant from the intestinal tract itself. For example, we have demonstrated that intestinal colonization with methicillin-resistant *Staphylococcus aureus* (MRSA) can result in remote surgical site infections in the absence of direct wound contamination, proposing a “Trojan Horse Hypothesis” to explain the occurrence of SSI. Here, MRSA becomes internalized by gut neutrophils and is carried silently through the blood stream – without causing sepsis or bacteremia – to surgical wounds where it is then released and can cause infection^66^. Additionally, we have shown that exposure to antibiotics and a Western diet (i.e. high fat, low fiber) predisposes mice to SSIs with polymicrobial organisms that are present in the gut, as revealed through amplicon sequence variant analysis. Notably, treatment with oral Pi-PEG, a phosphate-rich polymer, protected against SSI by gut-derived pathogens without changing the composition of the gut microbiota, suggesting that it acts during microbial transit from gut to wound. Pi-PEG is proposed to work in this model by suppressing bacterial virulence expression without affecting bacterial growth, thus supporting the function of the microbiome over the course of surgical injury^67^. Taken together, these findings challenge the traditional conceptual framework of post-operative infection that suggests that SSI can only develop via an external contamination event. Rather, this proposes that the gut microbiota – often perturbed by various selective pressures such as antibiotics and diet – can be a reservoir of potential pathogens that can cause infectious complications at remote sites. However, the extent to which internal versus external pathogen sources contribute to distant infection remains to be clarified.

### Clinical care and the gut microbiota

Many of the clinical interventions intended to promote recovery after traumatic injury or surgical stress in critically ill patients (e.g. antibiotics, artificial nutrition) can lead to further dysregulation in the gut ecosystem, leading to a vicious cycle of inflammation and organ failure^68^. Here, we describe how interventions and treatments intended to support patients through critical illness can instead drive further intestinal dysbiosis.

#### Antibiotics

Antibiotics are a mainstay for both infection prophylaxis in surgical patients, and in the treatment of infection-related complications in post-operative and trauma patients. While their use is often unavoidable, it can result in significant perturbations of the native gut flora. Given that the molecular targets of antibiotics are highly conserved across bacterial species, necessary antibiotic exposure can unfortunately result in collateral damage to commensal microbes, disrupting gut microbial ecology and providing opportunities for the enrichment of pathogenic organisms. Studies of healthy adult humans have demonstrated that the administration of broad-spectrum antibiotics leads to a rapid reduction in alpha diversity that only partially recovers over the course of weeks to months^69,70^. This is accompanied by the loss of critical metabolic functions, with multiple studies demonstrating that antibiotic administration is associated with decreased carbohydrate fermentation and bile acid metabolism^71,72^. In intensive care unit (ICU) patients, these effects are only amplified. A prospective study of ICU patients used multi-omics factor analysis to demonstrate the effects of antibiotic treatment and demonstrated a loss of overall diversity, marked depletion of anaerobic communities, and fungal expansion. This study also observed a decrease in fecal SCFA concentrations in critically ill and antibiotic-treated patients, and SCFA levels were inversely proportional to fungal copies^73^. Furthermore, the indiscriminate use of antimicrobials can drive the emergence of antibiotic resistance and expression of previously latent virulence factors as shown by our group^74^.

Ultimately, these antibiotic-induced alterations to gut microbiota structure and function allow for the rise of a gut pathobiome that leads to an increased susceptibility to infection-related complications and poor outcomes in critically ill patients. In a retrospective observational study of critically ill patients, early administration of anti-anaerobic antibiotics was associated with decreased infection-free and overall survival, which correlated with domination of the gut microbiota by *Enterobacteriaceae* ^24^. Additionally, a large multicenter study found that patients who were exposed to antibiotics during prior hospitalization were at 65% increased risk for developing sepsis within 90 days of discharge^75^. While the use of antimicrobials in the treatment of infection is often unavoidable, their unintended consequences on the intestinal microbiota may contribute to recurrent infections and poor outcomes. Taken together, these studies highlight the importance of careful antimicrobial stewardship to minimize collateral damage to the native gut flora^76^.

#### Artificial Nutrition

The use of artificial nutrition is another aspect of critical care medicine that can perturb the gut microbiota of patients in a way that leaves them susceptible to infectious complications^77^. The impact of diet on the gut microbiota is well-established, and a Western diet (i.e. high fat, low fiber) causes changes in gut microbiota composition and function in humans within a single day. Compared to individuals fed a plant-based, high-fiber diet, those fed a Western diet experienced a loss of biodiversity, lower concentrations of SCFAs, and higher concentrations of secondary bile acids, which in turn inhibit the growth of health-promoting phyla including *Bacteroidetes* and *Firmicutes* ^78^. In the ICU setting, enteral nutrition often consists of sterile, chemically defined formulas that lack any dietary fiber and differ dramatically from the plant-based diets that are known to support more resilient, health-promoting gut flora^79^. Additionally, many enteral feeds contain emulsifiers that have been linked to a reduction in diversity and SCFA production^80,81^. Furthermore, critically ill patients often experience frequent interruptions in enteral nutrition for numerous reasons (e.g., intestinal surgery, slow return of bowel function, vasopressor requirements). As a result, these patients may rely on total parenteral nutrition (TPN) to meet their caloric needs. While this provides nutrients to host tissues, it may inadvertently deprive microbes within the gut environment of key nutrients needed for survival. This situation may motivate certain microbes in the gut to invade deeper host tissues to obtain nutrition and lead to intestinal inflammation. The consequences of delivering nutrients exclusively into the systemic circulation via TPN have been examined in several preclinical models and human studies. TPN has been shown to cause a reduction in microbial diversity and in particular, domination by *Proteobacteria*, which are known to survive in nutrient-scarce states^82,83^. While artificial nutrition is often necessary in the management of critically ill patients, it is important to bear in mind that it can have a profound impact on the patient’s gut microbiota that may result in increased susceptibility to infectious complications and other adverse outcomes.

#### Other Medications

Patients are exposed to a number of other common medications throughout their clinical course that can have under-appreciated but significant impacts on the gut microbiota. Post-operative care often necessitates the use of opioids (e.g. morphine), which in mouse models can lead to expansion of communities of pathogenic bacteria, increase bacterial translocation, and induce a more virulent phenotype of *P. aeruginosa*, leading to increased risk of infectious complications and sepsis^84,85^. NSAIDs are also frequently used for analgesia or secondary prevention of cardiovascular events, in the case of aspirin. As with other drugs, their administration causes changes in the composition of the microbiota to favor an abundance of taxa that have metabolizing capability for NSAIDs, which includes gram-negative and pro-inflammatory strains^86–88^. Additionally, many patients may be chronically on proton pump inhibitors (PPIs) or may be started on them for stress ulcer prophylaxis during hospitalization. PPI use has been shown to result in compositional changes in the fecal microbiota, including an over-representation of oral bacteria as well as an increase in potentially pathogenic *Enterooccus, Streptococcus, Staphylococcus, and E. Coli* ^89^. Importantly, these microbiota changes are known to predispose patients to complications such as *C. difficile* infections^90^. Moreover, in the critical care setting, patients with profound vasoplegia are often administered stress dose corticosteroids. Corticosteroids have been shown to alter intestinal microbiota composition and decrease colonocyte mucus production, which may ultimately leave the host more vulnerable to infection. It is important to consider the impact that these relatively routine medications can have on the gut microbiota, as consequent compositional and functional changes in the microbiota can have under-recognized implications in the patient’s overall disease course and recovery trajectory.

### The rise of the pathobiome and associated functional changes

As described above, the physiologic stress of critical illness and its treatments disrupt the gut microbiota, driving a shift from a balanced, commensal community to one dominated by pathogenic, often drug-resistant strains^68,74,91,92^. Studies have shown dramatic changes to both the composition and function of the gut microbiota, with a loss of 90% of the normal anaerobic flora within the first six hours of a critical insult^68,93^. There is a relative depletion of obligate anaerobes such as *Firmicutes* and *Bacteroidetes*, and over time, there is an overgrowth of pathobionts including *Proteobacteria, Staphylococcus, Enterococcaceae*, as well as fungal species such as *C. albicans*. Several prospective observational studies have captured these pathogens dominance in association with a striking absence of microbial diversity in ICU patients^74,93–96^. For example, previous work from our laboratory has demonstrated that ICU patients harbor multidrug-resistant ultra-low-diversity microbial communities consisting of as few as 2 to 4 pathogens^74^. Another group compared the microbiota data of 115 ICU patients to that of healthy controls from the American Gut Project. They similarly found that critical illness is associated with the loss of health-promoting commensal microbes, and the degree of intestinal dysbiosis correlates with length of ICU stay^95^. These alterations to the gut microbiota have been implicated in the development of infectious complications and correlated with increased mortality in critically ill patients, as obligate anaerobes play a central role in inhibiting the overgrowth and translocation of pathogenic microbes. The loss of these commensal bacteria reduces colonization resistance, creating an environment that favors the emergence of the pathobiome.

With the compositional changes of the microbiota associated with critical illness comes a multitude of alterations in its functional output. Recent technological advances – particularly in high-throughput mass spectrometry and nuclear magnetic resonance spectroscopy – have allowed the field of metabolomics to expand rapidly over the past few decades. Metabolomics provides a more comprehensive way to study the functional changes of the gut microbiota in response to major physiologic perturbations and has helped elucidate the bi-directional relationship between host and microbiota. Several studies have shown that microbial metabolic processes, including production of SCFAs and other small metabolites by commensal bacteria through fermentation of carbohydrates and amino acids and bile acid dehydroxylation by colonic flora, become disrupted in critically ill patients. One group found dysregulated bile metabolism as well as reduced fecal excretion of SCFAs in critically ill children compared to healthy controls and associated increased fecal butyrate levels with days free of intensive care^97^. These results are consistent in adults, as several groups have shown that adult ICU patients exhibit similar decreases in levels of SCFAs that occurs immediately after severe insults and sustained long term^68,96,98^. Metabolites from commensal microorganisms play important physiological roles in the host – serving as energy sources, exerting immunomodulatory effects, and contributing to intestinal mucosal integrity – and their depletion in the setting of critical illness undoubtedly predisposes patients to a variety of complications. Thus, understanding the functional changes associated with the rise of the pathobiome is key in contextualizing how the microbiota can influence the trajectory of a patient’s disease progression or recovery.

The reasons behind the profound compositional and functional shifts in the intestinal microbiota in response to physiologic stress and critical illness remain poorly understood. One hypothesis is that these changes may represent an adaptive response, whereby the gut microbiota enters a quiescent, hibernation-like state to conserve energy in anticipation of nutrient deprivation. This concept of microbial dormancy is a well-established survival strategy observed across diverse ecosystems – including permafrost, deserts, deep soils, and even the human body – that allows organisms to endure harsh conditions^99–102^. Within the human body, certain infectious bacteria respond to stress by forming persister cells – non-dividing, metabolically dormant subpopulations – that remain inactive until conditions become more favorable, at which point they can reemerge and cause disease^103–105^. Beyond conserving scarce resources, this dormant state may also serve to minimize the bioburden of the gut microbiota to prevent further antigenic challenge to the host upon whom its survival may depend. However, modern medical interventions as discussed previously (e.g., prolonged hospitalization, antibiotics, artificial nutrition) may disrupt this adaptive response, as the hibernating native microbiome becomes supplanted by a pathobiome that can not only hinder recovery by driving immunosuppression but also contribute to new complications that compound the deleterious effects of the original insult.

### Reframing the current understanding of sepsis

By examining a patient’s disease course through this new lens which incorporates the role of host-microbe interactions in the development of various gut microbe-mediated complications, we propose a more comprehensive framework with which to approach sepsis – a term that has long been used to describe the clinical manifestations of infection as a systemic disorder. Clinically, sepsis is characterized by the rapid onset of systemic manifestations of organ failure in the setting of infection. Following surgery, this is usually due to some type of major infection such as burn-wound sepsis, deep organ space infection, an anastomotic leak, pneumonia, or a blood infection. Effective treatment of sepsis relies on its early recognition, prompt initiation of antimicrobial therapies, supportive care (e.g., fluid resuscitation and vasopressors to address hypoperfusion), and ultimately definitive source control via surgical intervention or drainage procedures^106^. Yet, despite advances in clinical management, sepsis is often described as a leading cause of morbidity and mortality worldwide^107,108^. However, there is a substantial proportion of patients who die *with* sepsis rather than *from* it, where the actual cause of death in patients with sepsis is often multifactorial, poorly defined, and at times obscured by the term “sepsis” itself^108–110^.

To more accurately understand the disease pathology of sepsis – beyond just an overwhelming systemic infection or immune dysregulation – it is important to recognize that most patients with sepsis die late in their disease course, often termed “late-onset sepsis^111^.” At this point, they have been subjected to the many selective pressures of critical care medicine that undoubtedly drive intestinal dysbiosis and are often colonized with highly virulent multi-drug-resistant pathogens that not only directly cause infectious complications but also contribute to systemic immune dysregulation. Thus, it becomes clear that sepsis is the result of a complex, dysregulated interaction between the host, the pathogen, and the microbiota that collectively drives the maladaptive inflammatory and metabolic cascades responsible for end-organ dysfunction. This also explains why current sepsis research and therapeutics often fall short, as they typically only target isolated components of a much more complicated, multifaceted process. Recognizing the role of the “interactome” in the development of sepsis offers insight into the heterogeneity of sepsis presentations and will ultimately inform new therapeutic strategies that focus on the preservation of the composition and function of health-promoting gut microbiota to drive recovery.

## Microbiota-directed therapies

The disease, its course, and its treatments all have implications on the gut microbiota which in turn shape the host response to disease and susceptibility to gut microbe-mediated infectious complications as described above. In light of this, there have been attempts to therapeutically modulate the gut microbiota with the intention of altering the disease course, preventing complications, and promoting recovery. As it is currently understood, the gut environment (i.e., the “soil”) and its overall microbial composition (i.e., the “seeds”) are both essential to maintain overall homeostasis when facing a severe and prolonged catabolic stress such as following major surgery or during critical illness. Two general approaches to modulate and enhance the gut in anticipation of such stress have been widely employed: provide the proper microbiota-accessible substrate (prebiotics), and replete the gut microbiota with known beneficial bacteria when those “seeds” are missing (probiotics). A combination of the two (synbiotics) or attempts to supplement with gut microbiota-derived metabolites have also been tried. Additionally, recognizing that a whole may be greater than the sum of its parts in that both soluble and particulate members of the final composition are needed, an entire fecal microbiota transplant (FMT) from another host has been proposed. Finally, in contrast to these reactionary strategies to restore the gut microbiota after physiologic stress, dietary prehabilitation prior to surgery has emerged as a more proactive approach to fortify the gut microbiota in a way that prevents microbe-mediated complications. Here, we discuss the clinical applicability and potential limitations of each of these microbiota-directed therapies, and provide a summary of select clinical trials assessing their safety and efficacy (Table 1).

**Table 1.** Outcomes from select clinical trials of synbiotics, probiotics, and fecal microbiota transplantation.

Therapy Type	Trial	Patient Population	Intervention	Route	Outcomes
Synbiotic	Rayes et al.^142^	Liver transplant (*n*=95)	Fiber + live or inactivated *L plantarum* vs standard enteral feeds	NJ	Decreased post-op infection fiber + live (13%) and inactivated (34%) *L plantarum* compared to standard enteral feeds (48%)
	Russolillo et al.^143^	Elective extrahepatic bile duct resection (*n*=40)	Galacto-oligosaccharides + *B bifidum, S thermophilus, S salivarius, L acidophilus, L casei, L bulgaricus*	Unknown	Decreased infection rate (50% vs 25%) and overall morbidity rate (70% vs 50%) in synbiotic group compared to control
	Sommacal et al.^144^	Periampullary neoplasm resection (*n*=100)	Fructo-oligosaccharides + *L acidophilus, L rhamnosus, L casei, B bifidum*	Unknown	Decreased rates of post-op infection (26.1% vs 69.6%), non-infectious complications (26% vs 60.8%), and hospital LOS (12 vs 23) in synbiotic group compared to control group
	Yokoyama et al.^145^	Whipple (*n*=44)	Galacto-oligosaccharides + *L casei, B breve*	NJ	No difference in incidence of infectious complications
	Polakowski et al.^146^	Colorectal cancer (*n*=73)	Fructo-oligosaccharides + *L acidophilus, L rhamnosus, L casei, B lactis*	PO	Decreased post-op infection (2.8% vs 18.9%) as well as serum IL-6 and CRP levels in synbiotic group compared to control
Probiotic	Zhang et al.^147^	Colorectal cancer (*n*=60)	*B longum, L acidophilus, E faecalis*	PO	Decreased post-op infection (10% vs 33.3%) as well as serum endotoxin, IL-6, and CRP levels in probiotic group compared to control
	Liu et al.^148^	Colorectal cancer liver metastasectomy (*n*=150)	*L plantarum, L acidophilus-11, B longum-88*	PO	Decreased post-op infection, faster return of bowel function, and decreased post-op transaminitis in probiotic group compared to control
	Grat et al.^149^	Liver transplant (*n*=105)	*L lactis, L casei, L acidophilus, B bifidum*	Unknown	Decreased 30-day (4.8% vs 34.8%) and 90-day (4.8% vs 47.8%) post-op infection rates, decreased post-op transaminitis in probiotic group compared to control
	Consoli et al.^150^	Colon resection (*n*=66)	*Saccharomyces Boulardii*	PO	Decreased post-op infection (13.3% vs 38.8%) in probiotic group compared to control
FMT	Van Nood et al.^120^	Recurrent CDI	Single infusion of donor stool (*n*=42)	ND	Resolution of CDI in 81% of patients in FMT group vs 31% in vancomycin and 23% in vancomycin + bowel lavage group
	Feuerstadt et al.^151^	Recurrent CDI	SER-109/VOWST (purified Firmicutes spores) (*n*=182)	PO	Decreased rate of recurrence (12% vs 40%) in SER-109 group vs placebo control
	Dubberke et al.^152^	Recurrent CDI	RBX2660/REBYOTA (Enema-based FMT)	PR	Increased treatment success rate (87.5% vs 58.1%) in those who received RBX2660 + placebo vs 2 doses of placebo

### Prebiotics

Prebiotics are dietary substrates that promote the growth and activity of the gut microbiota. These include fermentable fiber, phenolics and phytochemicals, conjugated linoleic acid, oligosaccharides, and polyunsaturated fats. The byproducts of prebiotics, once broken down by bacteria within the gut microbiota, are mainly SCFAs which have a myriad of positive impacts on the host, as previously described^112^. Prebiotic supplementation has been shown to increase the concentration of commensal bacteria in the gut microbiota while simultaneously decreasing pathogenic strains in human studies^113^. Further, prebiotics have beneficial immune-modulating effects. In rodent models of septic shock, prebiotics have been found to maintain gut barrier integrity, attenuate endotoxemia, and reduce mucosal inflammation^112^. Compared to other microbiota-directed therapies such as probiotics and FMT, prebiotics are less technically challenging to prepare and possess a longer shelf-life, making them more cost-effective. Side effects, such as osmotic diarrhea and bloating, are minimal. However, one limitation of using prebiotics to prevent infection-related complications following surgery or during critical illness is that providing substrate alone to a depleted microbiota may not be enough to repopulate the gut with the functioning microbiota it needs. Alternatively, while it may seem intuitive that fiber supplementation is health-promoting, based on the type of fiber selected, there is a significant potential for selection of particular species or communities of bacteria that may even be pathogenic^114^.

### Probiotics

Probiotics are live microorganisms that are used as therapeutics to supplement the host microbiota. While empiric probiotics are commonly consumed by healthy individuals with the goal of health promotion and disease prevention, probiotics have also been studied for their ability to restore microbial balance and enhance host recovery after physiologic stressors such as surgery and critical illness where dysbiosis is common. For instance, one meta-analysis showed a significant reduction in overall rate of infections and incidence of ventilator-acquired pneumonia (VAP), demonstrating that probiotics have some potential for reducing infectious complications^115^. However, some pause should be taken prior to administration of probiotics, because even beneficial probiotic bacteria have demonstrated potential to behave as pathogens and disseminate in the critically ill host^116^.

Furthermore, the efficacy of probiotics is highly individualized, and often limited by colonization resistance in the host, in which the native microbiota prevents sustained mucosal engraftment of exogenous strains. Using metagenomics and metatranscriptomics, one group demonstrated that while probiotics transiently appear in stool after administration, actual gut mucosal colonization by probiotic strains varies markedly between individuals^117^. Persistence of probiotics in the mucosal microbiome is highly influenced by a variety of host and microbial factors, including baseline recipient immune activity and microbiota composition^118,119^. This variability in mucosal colonization resistance to probiotics presents a significant challenge in using probiotics predictably in clinical settings. In post-surgical and critical care patients, where the microbiota may be disrupted by antibiotics, inflammation, or nutritional deficiencies, probiotics hold theoretical promise for restoring barrier function, reducing infections, and modulating systemic inflammation^40^. yet, the lack of consistent consistent colonization and the potential for adverse events – particularly in immunocompromised or critically ill patients – underscore the need for caution as well as a need for further study. Probiotic formulations are currently unregulated as pharmaceuticals, and their live bacterial components can behave unpredictably under stress. Thus, moving forward, a more personalized, data-driven approach that involves selecting probiotic strains based on recipient-specific microbiome features and utilizing tools like metagenomics to predict compatibility and response may be warranted. This approach could transform probiotics from a one-size-fits-all empiric supplement into a more targeted therapeutic tool to drive recovery in post-surgical, trauma, and critical care patients.

#### Fecal Microbiota Transplant

Fecal microbiota transplantation (FMT) has garnered increasing attention as a possible treatment for a broad range of diseases and has been demonstrated to be particularly effective for refractory *C. difficile* diarrhea^120^. FMT involves transferring a complex, incompletely characterized microbial community from a carefully screened healthy donor to a recipient, with the ultimate goal of restoring key microbiota functions (e.g. colonization resistance, metabolite production) and reestablishing balance within the interactome. There have been several indications that FMT may have the ability to alter the host immune system via the gut microbiota with evidence in mice that FMT can increase production of butyrate and indole metabolites resulting in improved survival from lethal bacterial infection^16,116^. Furthermore, one promising avenue for the use of FMT in the setting of infection and critical illness is the use of an autologous FMT to expedite refaunation of the microbiota following antibiotic exposure^121^.

In clinical practice, the success of FMT in treating recurrent *C. difficile* colitis in particular has prompted exploration of its potential in treating a range of other conditions including graft-versus-host disease^122–124^, diabetes^125^, and inflammatory bowel disease^126–128^. Although some benefits have been observed, the efficacy of FMT in these settings has not matched the robust outcomes seen in its use as a treatment for *C. difficile* ^129^. Moreover, existing clinical trials vary widely in design and sample size, limiting the ability to draw any definitive conclusions about FMT’s broader therapeutic utility. Despite these limitations, current studies offer valuable insight into the challenges associated with FMT, particularly regarding its limitations, inter-individual variability in response, and concerns surrounding long-term safety. A major concern remains the potential transmission of pathogenic organisms through FMT. For example, there have been reports of multi-drug-resistant *E. Coli* bacteremia transmitted after FMT and other infectious complications^130–132^. While the American Gastroenterological Association has established a registry to monitor long-term outcomes following FMT, it is clear that further work is necessary to fully characterize and address the long-term risks associated with biotherapeutics such as FMT.

One of the core challenges with FMT is its nature as a biological “black box.” The mechanisms underlying its therapeutic effects remain poorly understood. It is unclear which bacterial strains are essential for efficacy, which microbial metabolites provide therapeutic benefit, or how the introduced community interacts with the recipient’s native microbiota. These interactions – which can be both symbiotic and antagonistic – may profoundly affect clinical outcomes. Moreover, the diversity of both donor and recipient microbiota influences the degree of engraftment,^125,126^ which likely contributes to the variable efficacy of FMT for indications other than *C. difficile*, given that the recipient microbiota may exert some level of colonization resistance in these cases. For example, the success of FMT in modulating glucose metabolism in patients with metabolic syndrome has been found to depend on the recipient’s baseline microbiota composition^133^. In addition to clinical variability, the development and commercialization of FMT present unique regulatory and manufacturing challenges. Unlike conventional pharmaceuticals, where composition and quality are reproducible and able to be tightly controlled, FMT involves culturing live organisms which has inherent inconsistency. Even under standardized growth conditions, organisms can change virulence expression, mutate, and gain or lose certain functions. As such, biotherapeutics require rigorous oversight and regulation to ensure both safety and consistency in clinical applications.

It is in this context that the field is increasingly focused on developing defined microbial consortia – rationally assembled communities of well-characterized bacterial strains designed to reestablish microbial balance and promote beneficial host outcomes in a variety of pathologies. Preclinical studies have supported this concept, as specific microbial consortia of symbiotic strains have been shown to successfully treat *C. difficile* as well as restore colonization resistance against vancomycin-resistant *E. faecium* in murine models^134,135^. In these efforts, understanding the interactome becomes paramount. Uncovering how individual microbes and their metabolic outputs interact with the host and with one another will be key to harnessing these systems for therapeutic use. Furthermore, the advent of metagenomic sequencing has further enabled efforts to determine compatibility of donor and recipient microbiota and personalize microbiota-directed therapies. In fact, some groups have identified species-specific engraftment patterns after FMT and have leveraged this to develop ways to predict donors that might optimize post-FMT recipient microbiota characteristics for specific diseases^136^. The ultimate goal would be to provide personalized microbiota-directed therapies tailored to an individual’s disease state as well as their resident microbiota.

### Dietary prehabilitation

Finally, dietary prehabilitation is an emerging preventative strategy designed to bolster the gut microbiota in anticipation of physiologic stressors such as surgery, with the ultimate goal of enhancing host resilience and improving post-operative outcomes. An individual’s baseline gut microbiota composition and its capacity to respond to surgical stress is increasingly recognized as a reflection of the host’s life history, including a multitude of social, environmental, and behavioral determinants of health. Social determinants such as diet quality, access to healthcare, early life exposures, and socioeconomic status are all intertwined and ultimately shape gut microbiota composition and function, which in turn affects immune responsiveness and thus vulnerability to post-operative complications. Recent work suggests that the gut microbiota possesses an “ecological memory” of all environmental exposures that can influence human health, termed the “exposome”^137^. As such, personalized, microbiome-informed prehabilitation offers a means to proactively optimize the gut ecosystem with the goal of modifying the effects of long-term exposures to various social determinants of health^138^. It is well-established that diet has a major influence on the gut microbiota and its response to physiologic stress, as described previously^139^. Our group has shown that seven days of pre-operative dietary prehabilitation with a high fiber, low fat diet was sufficient to reverse the deleterious effects of a high fat, low fiber Western diet on the intestinal microbiome and improves post-operative survival in a murine hepatectomy model^138^. We have additionally demonstrated that prehabilitation with high fiber, low fat diet can improve colonic anastomotic healing in mice^140^. Furthermore, a meta-analysis of five randomized-controlled trials in humans found that nutritional prehabilitation decreases the risk of post-operative complications in patients undergoing esophagectomy or gastrectomy for esophagogastric cancer^141^. Taken together, these findings suggest that prehabilitation is a promising strategy for promoting microbiome readiness for surgery and preventing microbe-related post-operative complications.

## Conclusions

**Figure 1.**
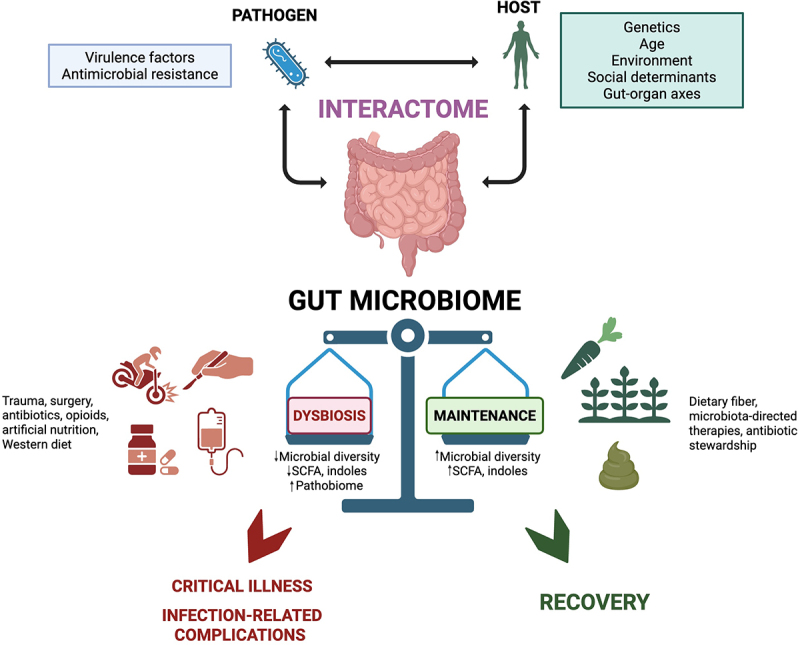
The interactome and its influence on disease progression versus recovery.

The gut microbiota plays a vital role in host recovery, immune modulation, and susceptibility to infection following physiologic stressors such as trauma, surgery, and critical illness. While microbiota-directed therapies including dietary prehabilitation, prebiotics, probiotics, and FMT show some promise, their clinical application remains limited by inconsistent efficacy and incomplete mechanistic understanding. As we continue to unlock the relationship of the gut microbiota to health and disease, it is becoming increasingly clear that outcomes are shaped by the interactome – the complex bidirectional dialogue between host, microbiota, and pathogen. Importantly, the extent to which the metabolome – a functional readout of host-microbiota interactions – is preserved in the setting of critical illness plays a central role in determining whether a patient who has been subject to a major physiologic insult progresses toward recovery, supported by a resilient gut microbiota, or deteriorates due to the effects of an expanding pathobiome. Looking ahead, further research should focus on elucidating the intricacies of microbial metabolism and interspecies cooperativity, with tools like metagenomic and metabolomic profiling offering pathways to precision microbiota-directed interventions, tailored to individual microbiota signatures. A deeper understanding of how the interactome (Figure 1) evolves throughout the course of illness and treatment will pave the way for more personalized, mechanistic approaches to microbiota-directed therapies in surgical and critical care settings.
